# The Lipid-Bound Apolipoprotein A-I Cysteine Mutant (N74C) Inhibits the Activation of NF-κB, JNK and p38 in Endotoxemic Mice and RAW264.7 Cells

**DOI:** 10.1371/journal.pone.0051327

**Published:** 2012-12-12

**Authors:** Yunlong Wang, Shulai Lu, Xinde Li, Na Du, Yunbo Sun, Jinyan Xing, Xinting Pan, Baosheng Chen, Zhimin Miao

**Affiliations:** 1 Gout laboratory, The Affiliated Hospital of Medical College Qingdao University, Shandong Provincial Key Laboratory Of Metabolic Diseases, Qingdao, China; 2 Stomatological Department, Qingdao Municipal Hospital, Qingdao, China; 3 Key Laboratory of Marine Drugs, Ministry of Education of China, School of Pharmacy, Ocean University of China, Qingdao, China; 4 ICU, The Affiliated Hospital of Medical College Qingdao University, Qingdao, China; 5 National Laboratory of Medical Molecular Biology, Department of Biochemistry and Molecular Biology, Institute of Basic Medical Sciences, Chinese Academy of Medical Sciences and Peking Union Medical College, Beijing, China; University of Pittsburgh, United States of America

## Abstract

Our previous studies showed that recombinant high-density lipoprotein (rHDL) rHDL74 exhibited higher anti-inflammatory capabilities compared to wild-type rHDL (rHDLwt), while rHDL228 showed hyper-proinflammation. In this paper, we further investigated the potential mechanisms involved in their different inflammatory functions using two models: endotoxemic mice and the RAW264.7 inflammation model. Our results showed that 24 h after the injection of lipopolysaccharide (LPS), mice treated with rHDL74 had a significant decrease in plasma CRP (P<0.01 vs. rHDLwt; P<0.01 vs. LPS), MCP-1 (P<0.05 vs. rHDLwt; P<0.01 vs. LPS) and CD14 (P<0.01 vs. LPS) compared with the mice treated with rHDLwt or the controls that received LPS only. Similar to our previous study, rHDL228 increased the plasma level of CRP (P<0.05 vs. LPS) and MCP-1 (P<0.01 vs. LPS). Our immunohistochemistry and western blot analysis showed that rHDL74 inhibited the activation of NF-κB in endotoxemic mice and JNK and p38 in the RAW264.7 inflammation model, while rHDL228 exacerbated the activation of NF-κB and ERK. In summary, our data suggest that rHDL74 exhibits higher anti-inflammatory activity by decreasing inflammatory factors and inhibiting the activation of NF-κB, JNK and p38, while rHDL228 appears to be hyper-proinflammation by increasing these inflammatory factors and aggravating the activation of NF-κB and ERK.

## Introduction

Lipopolysaccharides (LPS) are extremely strong stimulators of inflammatory reactions and are involved in the pathogenesis of sepsis and septic shock. The LPS molecule consists of four different parts, including lipid A, the inner core, the outer core and the O antigen [1], [2], [3]. Experiments with synthetic lipid A have shown that this part of the LPS molecule represents the toxic moiety [4]. After entering the bloodstream, LPS binds to LPS-binding protein (LBP), which helps in transferring LPS to its co-receptor CD14, leading to the formation of receptor clusters of Toll-like receptor 4 (TLR4), CD14, and other adaptors. This cluster results in the activation of the nuclear factor-κB (NF-κB), JNK, p38 and ERK pathways and the production of a cascade of pro-inflammatory cytokines, such as monocyte chemoattractant protein-1 (MCP-1), C-reaction protein (CRP), and tumor necrosis factor α (TNF-α), which mediate the cellular inflammatory response to Gram-negative infection [5], [6]. Moreover, LPS can be neutralized when combined with plasma high-density lipoprotein (HDL) [7], [8], [9], [10].

Apolipoprotein A-I (apoA-I), the major protein component of HDL, is known to play an important role in cardioprotection against atherosclerosis via anti-inflammatory and anti-oxidative activity [11], [12], [13]. Apolipoprotein A-I_Milano_ (apoA-I_Milano_) and apoA-I_Paris_, two natural cysteine mutants of apoA-I with dimers as their effective forms, have been shown to have enhanced cardiovascular protective activities [14], [15], [16]. Based on the Edmundson wheel [17], seven cysteine mutants of apoA-I were designed and constructed in our laboratory with mutant positions similar to apoA-I_Milano_ on the α-helices in our previous study, including cysteine variants of apoA-I at residues 52 (S52C), 74 (N74C), 107 (K107C), 129 (G129C), 195 (K195C), and 228 (S228C) [18], [19]. These variants exhibited different structural features regarding their secondary structure and biological activities of binding lipids, anti-peroxidation, and promoting cholesterol efflux from THP-1 macrophages [18]. In addition, our previous studies showed that rHDL74, which was reconstituted by mixing apoA-I (N74C) with dipalmitoyl phosphatidylcholine, had increased anti-inflammatory capability in an LPS-induced endotoxemic mouse model compared with wild-type apoA-I (apoA-Iwt) and apoA-I_Milano_, while rHDL228 showed hyper-proinflammation by exacerbating LPS-induced endotoxemia in mice [19]. In this study, we used the endotoxemic mouse model and the RAW264.7 inflammation model to further investigate the different effects of rHDL74 and rHDL228 on inflammation.

**Table 1:** Experimental groups used in the LPS and rHDL injection study.

Group	Injection	LPS (mg/kg)	rHDL(mg/kg)	N
Saline	300 µl Physiologic saline	−	−	6
LPS	LPS	4	−	6
rHDLwt	LPS/rHDLwt	4	80	4
rHDL74	LPS/rHDL74	4	80	4
rHDL228	LPS/rHDL228	4	80	4

## Materials and Methods

### Materials

LPS (from *Escherichia coli* 055:B5) and 1,2-dipalmitoyl-sn-glycero-3-phosphocholine (DPPC) were purchased from Sigma. The bicinchoninic acid protein assay kit, Detoxi-Gel™ endotoxin-removing gel, horseradish peroxidase-conjugated secondary antibodies and chemiluminescence reagents were purchased from Pierce. ELISA kits were purchased from R&D. Male BALB/c mice (18–20 g) were purchased from the laboratory animal department of Peking Union Medical College. RAW 264.7 macrophages were purchased from ATCC. DMEM was purchased from HyClone. A polyclonal antibody against NF-κB p65 was purchased from Santa Cruz Biotechnology. Two-Step IHC Detection Reagents were purchased from Zhongshan Goldenbridge Biotechnology Company. The antibodies used in western blot analysis, including anti-phospho-Erk1/2, anti-Erk1/2, anti-phospho-p38 MAP kinase, anti-p38 MAP kinase, anti-phospho-Jnk, and anti-Jnk, were purchased from Cell Signaling, and anti-beta-actin was purchased from Sigma. All animal experiments were approved by the animal care committee of the Affiliated Hospital of Medical College Qingdao University.

**Figure 1:**
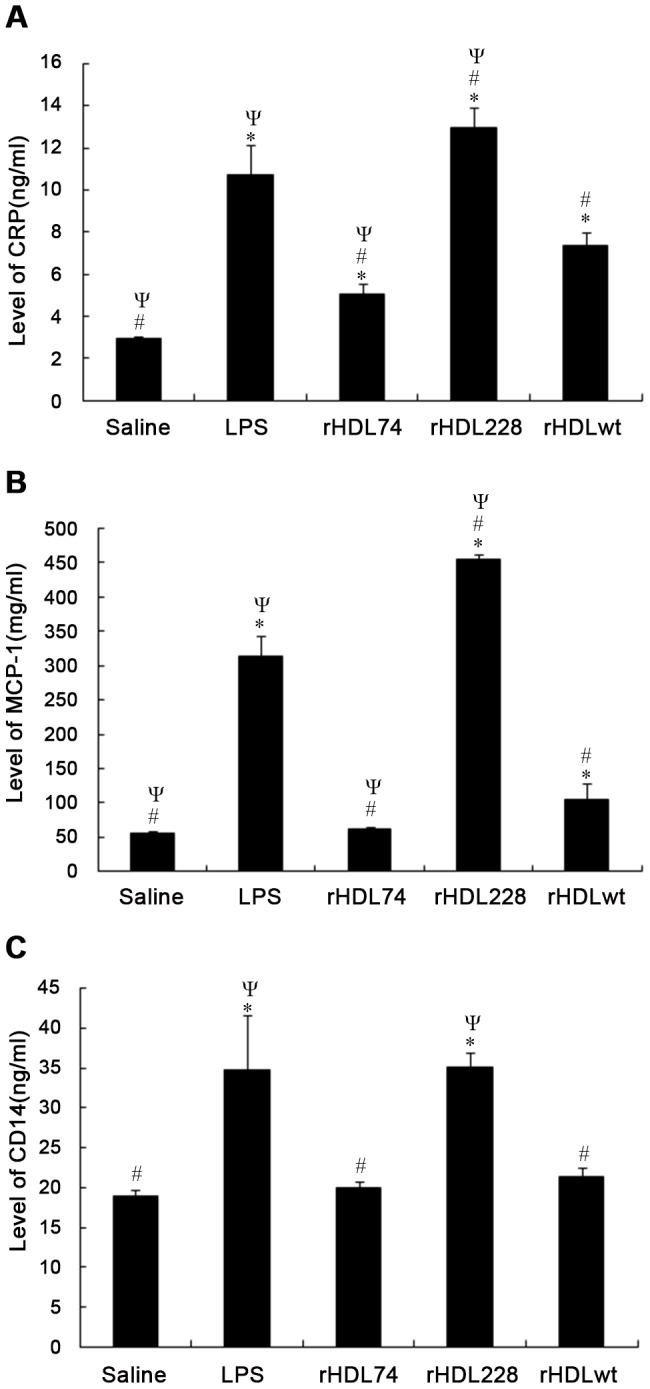
CRP, MCP-1 and CD14 levels at 24 h after LPS injection. Control: without any treatment. LPS, rHDLwt, rHDL74 and rHDL228 represent the groups that were treated with LPS, LPS+rHDLwt, LPS+rHDL74 and LPS+rHDL228, respectively. Compared with the LPS group, mice treated with rHDL74 showed significantly decreased levels of CRP, MCP-1 and CD14. However, mice receiving rHDL228 exhibited higher levels of these factors. In addition, compared with rHDLwt, rHDL74 significantly reduced the level of CRP and MCP-1. To confirm our experimental results, we performed three independent experiments. A: Levels of CRP. B: Levels of MCP-1. C: Levels of CD14. ^*^P<0.05 vs. control; ^#^P<0.05 vs. LPS; ^Ψ^P<0.05 vs. rHDLwt. Error bars indicate the SD.

### Preparation of Recombinant HDLs

The expression, purification, and endotoxin removal of recombinant proteins, including apoA-Iwt, apoA-I (N74C), and apoA-I (N228C), and the construction of recombinant HDLs were performed as described previously [18], [19]. The purified proteins were lyophilized and stored at −70°C.

**Figure 2:**
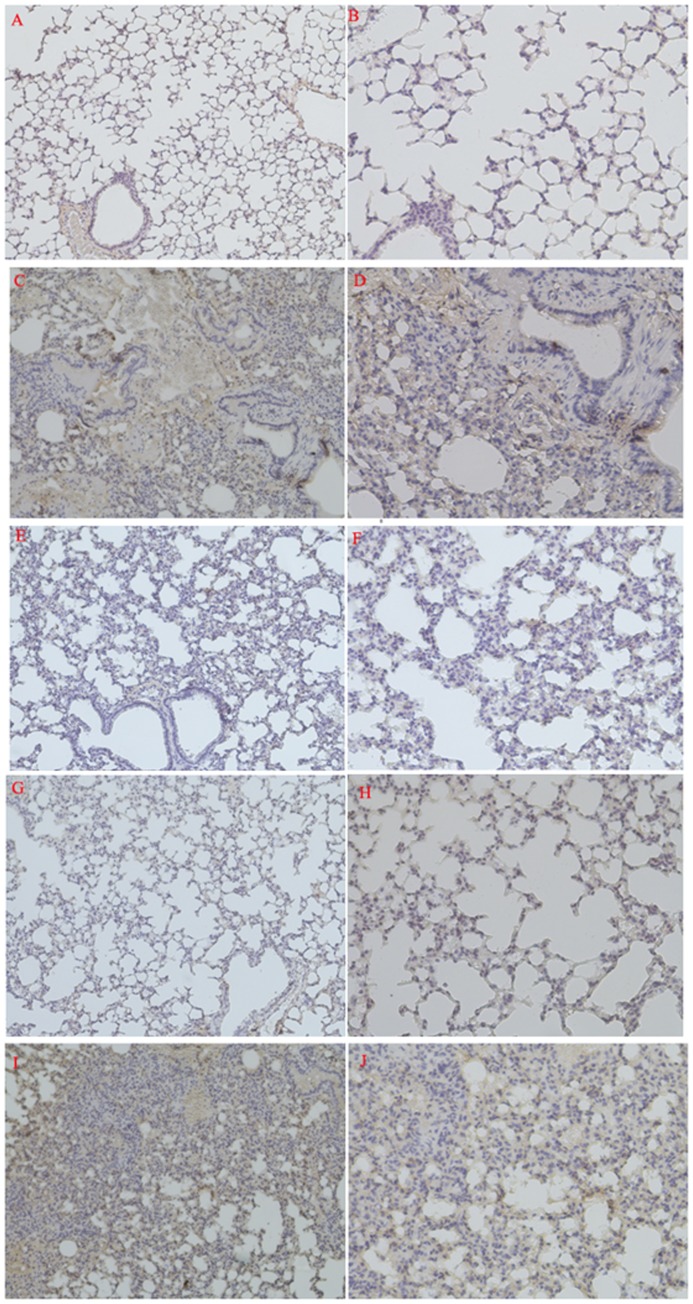
Photomicrographs of representative immunostained sections of lung. (A, C, E, G, I: magnification ×100; B, D, F, H, J: magnification ×200). A, B: Saline group treated with physiologic saline only. C, D: LPS group without any other treatment. E, F: LPS group treated with rHDLwt. G, H: LPS group treated with rHDL74. I, J: LPS group treated with rHDL228. Compared with the saline group (A, B), mice in the LPS group (C, D) exhibited a higher expression of NF-κB p65, as shown by the arrows. Compared with the LPS group, rHDLwt (E, F) significantly decreased the expression of NF-κB p65. There was almost no positive expression of NF-κB p65 in the mice in the LPS group treated with rHDL74 (G, H). Mice receiving rHDL228 (I, J) showed aggravated expression of NF-κB p65. To confirm our experimental results, we performed three independent experiments.

### Treatment of Septic Mice with Different Recombinant HDLs

The treatment of the in vivo LPS-induced endotoxemic mouse model with different rHDLs was performed as previously described [5], [20]. The animals were divided into 5 experimental groups as shown in Table 1, where N denotes the number of animals in each group. Two control groups were used in this study: an LPS group, in which mice received only LPS by tail vein injection (4 mg/kg) to induce endotoxemia; and a saline group, in which mice received only 300 µl of physiologic saline as a control. In the experimental rHDL groups, LPS was injected through the tail vein, and after the anal temperature increased by 0.5°C (10 min 54 s ±30 s after LPS injection), each of the rHDLs (80 mg/kg) prepared above was slowly injected into the mice through the tail vein within 60 s. Twenty-four hours after injection, the mice were euthanized, and plasma was isolated by centrifugation at 12,000 g for 10 min and stored at −80°C until analysis. To confirm our experimental results, we performed three independent experiments. All animal experiments were approved by the animal care committee of the Medical College of Qingdao University.

**Figure 3:**
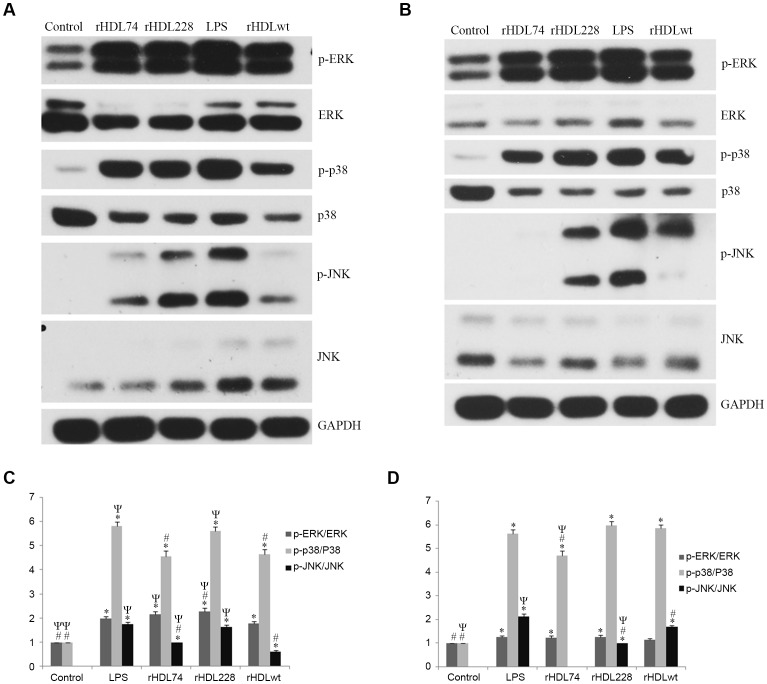
Western blot analysis of the inflammation signaling pathway in RAW264.7 cells induced by LPS. To investigate the effect of different rHDLs on inflammation, we examined the signaling pathway of the RAW264.7 inflammation model treated with different rHDLs. Our results showed that rHDL74 and rHDLwt significantly decreased the activation of p38 and JNK (p<0.05 vs. LPS), while rHDL228 aggravated the activation of ERK (p<0.05 vs. LPS). Control group: RAW264.7 cells without any treatment; LPS group: RAW264.7 cells induced by LPS; rHDL74 group: RAW264.7 inflammation cells treated with rHDL74; rHDL228 group: RAW264.7 inflammation cells treated with rHDL228; rHDLwt group: RAW264.7 inflammation cells treated with rHDLwt. To confirm our experimental results, we performed three independent experiments. A. Western blot analysis at 12 h after rHDL treatment. B. Western blot analysis at 24 h after rHDL treatment. C. The gray value analysis of western blot bands with Image J at 12 h after rHDL treatment. D. The gray value analysis of western blot bands with Image J at 24 h after rHDL treatment. ^*^P<0.05 vs. control; ^#^P<0.05 vs. LPS; ^Ψ^P<0.05 vs. rHDLwt. Error bars indicate SD.

### Detection of Plasma Levels of CRP, CD14 and MCP-1

The plasma levels of CRP, CD14 and MCP-1 were measured by ELISA kits according to the manufacturer’s instructions.

### Immunohistochemical Staining and Assessment in Septic Mice Treated with Different Recombinant HDLs

Twenty-four hours after LPS injection, the lungs were isolated and fixed in 10% formaldehyde solution at room temperature and routinely processed for paraffin embedding. Immunohistochemical staining was carried out on tissue microarray sections according to the manufacturer’s instructions. Positive and negative immunohistochemistry controls were routinely used.

### The Construction of the RAW264.7 Inflammation Model and the Effect of Different Recombinant HDLs on the RAW264.7 Inflammation Model

RAW 264.7 macrophages were maintained in DMEM supplemented with 10% (v/v) fetal bovine serum, 100 U/ml penicillin and 100 µg/ml streptomycin at 37°C (5% CO_2_). Exponentially growing RAW264.7 cells were digested with 2.5 mg/ml trypsin and suspended in DMEM to a concentration of 2×10^5^ cells/ml, and the total number of cells was determined with a hemocytometer. Subsequently, the cells were plated in 6-well flat-bottomed microculture plates (2 ml/well) and cultured at 37°C in a 5% CO_2_ atmosphere for 4 h. The cultures were washed to remove non-adherent cells and then incubated with 2 ml of DMEM supplemented with 10% (v/v) fetal bovine serum, 100 U/ml penicillin and 100 µg/ml streptomycin for an additional 20 h.

The cell plates were randomly divided into three incubation periods after treatment with rHDL: 12 h, 24 h and 48 hs. For each incubation period, there were five groups: control group: served as control without any treatment; LPS group: received only LPS to induce inflammation; and rHDL74, rHDL228 and rHDLwt groups: received LPS and then treated with rHDL74, rHDL228 and rHDLwt, respectively. The culture medium of all groups was replaced with FBS-free DMEM for 30 minutes to allow cells to adjust. Then, 1 µg/ml LPS was added to all groups except the control group to induce an inflammation model. After 24 h of stimulation with LPS, purified rHDL was added to each group at a final concentration of 5 µg/ml.

### Western Blot Analysis of the Inflammation Signaling Pathway in RAW264.7 Cells

Cells were scraped into 100 µl lysis buffer (50 mM Tris-HCl, pH 6.8, 2% SDS, and 10% glycerol) supplemented with 1 µl (100×) protein inhibitor cocktail at 12 h, 24 h and 48 h after treatment with different rHDLs, and samples were boiled at 100°C for 10 min and clarified by centrifugation at 12,000 rpm for 10 min at room temperature. Protein concentrations were determined by the BCA method. Equivalent protein amounts were separated on 10% SDS-polyacrylamide gels and transferred to Immobilon-P polyvinylidene fluoride membranes. The blots were then hybridized with specific primary antibodies, and antigen-specific signals were detected using horseradish peroxidase-conjugated secondary antibodies and chemiluminescence. Gel analyses were performed to determine the gray value of the western blot bands using Image J.

### Statistical Analysis

SPSS17.0 software for Windows (SPSS Inc., USA) was used for statistical analysis, except for western blot results. The values were expressed as the mean ± SD, and differences between groups were examined for significance using a one-way ANOVA for multiple comparisons. Two-tailed t-tests were performed with Excel for the analysis of western blot results. P<0.05 was considered to be statistically significant.

## Results

### Different Effect of rHDLs on the Expression of Inflammatory Cytokines in the Plasma of Endotoxemic Mice

To determine the effect of the three rHDLs on endotoxemic mice, we evaluated the plasma levels of CRP, MCP-1 and CD14 by ELISA. As shown in Figure 1A, 24 h after LPS injection, mice receiving rHDLwt exhibited significantly lower levels of plasma CRP (7.33±0.89 µg/ml, P<0.001) compared with the LPS group (11.02±1.58 µg/ml). Compared to mice treated with rHDLwt, mice treated with rHDL74 also showed a significant decrease in levels of plasma CRP (rHDL74∶4.96±0.72 µg/ml, P = 0.006 vs. rHDLwt). Interestingly, mice treated with rHDL228 had much higher plasma levels of CRP (rHDL228∶12.85±1.35 µg/ml, P = 0.014) compared with the LPS group. The mice treated with rHDLwt and rHDL74 exhibited a significantly decreased level of MCP-1 (rHDLwt: 103.43±26.37 pg/ml, P<0.001 vs. LPS; rHDL74∶62.20±2.88 pg/ml, P<0.001 vs. LPS) compared with the LPS group (309.65±33.11 pg/ml) (Fig. 1B). Mice receiving rHDL74 also had decreased plasma levels of MCP-1 (P = 0.013) compared to mice treated with rHDLwt. The level of MCP-1 in mice treated with rHDL74 was close to the saline group (rHDL74∶62.20±2.88 pg/ml; saline: 57.00±0.70 pg/ml, P = 0.73). However, mice treated with rHDL228 had significantly higher levels of MCP-1 compared to those treated with LPS only (rHDL228∶453.22±9.19 pg/ml, P<0.001 vs. LPS). We also found that mice treated with rHDL74 and rHDLwt had significantly reduced levels of plasma CD14 compared with the LPS group (rHDL74∶20.00±0.95 ng/ml, P<0.001 vs. LPS; rHDLwt: 21.45±1.52 ng/ml, P = 0.001 vs. LPS), but we did not observe any significant differences between rHDL74 and rHDLwt in reducing plasma CD14 (P = 0.702) (Fig. 1C).

### Immunohistochemical Detection of NF-κB p65 in Lung Tissue

As shown in Figure 2, compared with the saline group (Figs. 2A and 2B), the lungs of mice receiving only LPS (Figs. 2C and 2D) had significant pathological changes: 1) congestion; 2) broadening of pulmonary interstitial tissue; 3) leukocyte infiltration, including monocytes and neutrophils; and 4) high NF-κB p65-positive expression. As shown in Figures 2E and 2F, the mice treated with rHDLwt exhibited only low NF-κB p65-positive expression compared with the saline group. In addition, the ability of rHDL74 to block the LPS-induced NF-κB pathway in this septic mouse model was strongly supported by its immunohistochemical results (Figs. 2G and 2H), which were close to the saline group, and there was almost no positive expression of NF-κB p65 in the rHDL74 group. However, in lung sections (Figs. 2I and 2J) from mice treated with rHDL228, we observed aggravated NF-κB p65 expression compared with the LPS group.

### Western Blot Analysis of the Inflammation Signaling Pathway in the RAW264.7 Inflammation Model Treated with rHDL

To investigate the potential mechanisms of different rHDLs on inflammation, we examined the signaling pathway in the RAW264.7 inflammation model treated with rHDLs. As shown in Figure 3, at 12 h after treatment with rHDLs, RAW264.7 cells treated with rHDL74 or rHDLwt had a significant decrease in p38 activation compared with the LPS group. rHDL74 and rHDLwt also showed decreased JNK activation compared with the LPS group. Moreover, rHDL228 aggravated the activation of ERK. At 24 h after treatment with rHDLs, RAW264.7 cells treated with rHDL74 had a significant decrease in p38 activation compared with the LPS and rHDLwt groups. More importantly, rHDL74 strongly inhibited the activation of JNK; we observed little phosphorylation of JNK. We found that rHDL228 significantly decreased the activation of JNK. Additionally, although there was no statistical significance (p = 0.06>0.05 vs. LPS), rHDL228 aggravated the activation of p38.

## Discussion

Our previous studies showed that recombinant HDL74 exhibited higher anti-inflammatory and anti-angiosclerotic capabilities, while rHDL228 showed hyper-proinflammation. In this study, we sought to identify the different mechanisms of these two rHDLs in inflammation. Our results showed that rHDL74 has protective effects in endotoxemic mice and the RAW264.7 cell inflammation model, which was supported by the following data: 1) a significant reduction of CRP, MCP-1, and CD14 in endotoxemic mice (p<0.001); and 2) a significant inhibition of the activation of NF-κB in endotoxemic mice and JNK and p38 in RAW264.7 cells. In contrast, rHDL228 elevated the plasma level of CRP, MCP-1 and CD14 and aggravated the activation of NF-κB and ERK.

The protective effects of HDL following LPS exposure have been well documented. Epidemiological studies have shown that HDL levels are inversely correlated with the outcome of endotoxic shock [21], [22], [23]. Additionally, the systemic administration of reconstituted HDL in human volunteers down-regulated CD14 on monocytes and attenuated pro-inflammatory mediators caused by small doses of intravenous LPS [24]. HDL also protects against LPS-induced inflammation and lethality in experimental animal models [5], [6], [7]. It has been found that a twofold increase in plasma HDL in human apoA-I transgenic mice enhances the binding of intraperitoneally administered LPS to HDL, reduces plasma TNF-α levels and improves the survival rate compared to control mice [5]. Likewise, an intravenous injection of reconstituted HDL increased survival in mice injected with LPS and in rabbits challenged with live *Escherichia coli* [25]. Several in vitro studies have demonstrated that apoA-I and sphingosine-1-phosphate (S1P), a sphingolipid associated with lipoproteins, especially HDL, inhibit LPS-induced inflammatory responses. Reconstituted HDL and an apoA-I mimetic peptide reduced the LPS-induced expression of endothelial cell adhesion molecules in vitro [26], [27]. S1P significantly reduced pulmonary vascular leakage and inflammation in a murine model of LPS-induced acute lung injury [28]. Taken together, these studies indicate a therapeutic function of rHDL. Our study is consistent with previous findings in which we also observed similar therapeutic functions of rHDLwt. NF-κB, JNK, p38 and ERK were the most important factors playing a pivotal role in mediating inflammatory responses to a variety of signals, including inflammatory cytokines [29]. In our study, rHDL74 exhibited a higher capability to inhibit the activation of NF-κB, JNK and p38 compared to rHDLwt, while rHDL228 aggravated the activation of NF-κB and ERK. Thus, our data indicate that the different mechanisms of rHDL74 and rHDL228 in inflammation were associated with the regulation of inflammatory factors and the activation of NF-κB, JNK, p38 and ERK. In summary, compared with rHDLwt, rHDL74 has higher anti-inflammatory properties by decreasing inflammatory factors and inhibiting the activation of NF-κB, JNK, and p38, whereas rHDL228 shows hyper-proinflammation by increasing these inflammatory factors and aggravating the activation of NF-κB and ERK.
